# 

**Published:** 1918-08-10

**Authors:** 


RATES OF SUBSCRIPTION :

Twelve months, 10/10; Six months, 5'5; Three months, 2/9, post free to any address in the United Kingdom. Abroad, Twelve months, 13/-; Six months, 6/6; Three months, 3/3, post free. **THE HOSPITAL "Index" to Volume LXI., October 19 7 to March 1918 is now ready, price 3½d. per copy, post free.** Address The Manager, THE HOSPITAL, "The Hospital" Building, 28/29 Southampton Street, Strand, London, W.C. 2.

**IMPORTANT.**

### BURDETT'S HOSPITALS & CHARITIES.

The new edition of this Authoritative Year Book will be published shortly, and as, in consequence of the paper restrictions, the issue must necessarily be limited, all orders should be placed without delay.

**Price 12/6** **By Post 13/-**

Any Bookseller can supply the book, or it may be obtained directly from the Publishers—

THE SCIENTIFIC PRESS, LTD., **28/29 Southampton Street, Strand, London, W.C. 2.**

Mr. Henry James Helm, of Bromley, late of the Inland Revenue Laboratory, left £100 to the Sea-Bathing Hospital, Margate, and part of the residue of his estate to the Urban Council of Padiham for sending poor persons to convalescent homes.

### Passing Events and Topics.

### Infants' Ration Books.

The Ministry of Food announces that in order to prevent any delay in issuing an infant's ration book, Food Control Committees have been instructed to issue an emergency card in cases where registration of birth is unavoidably delayed. This card will bear enough coupons to provide the infant's weekly ration during the probable interval between birth and date of registration. Such card should be exchanged for an infant's ration book as soon as the birth certificate is obtained. Application for the emergency card should be made on Form N. 30, suitably modified, and must be countersigned by the doctor or midwife in charge of the case. The form can be obtained from any Local Food Office, and, after being duly filled in, can be presented by anyone acting on behalf of the parents.

### The Prevailing Epidemic.

The whole of Europe has been visited by an acute, infectious, catarrhal fever which has been popularly called influenza. Its most striking features are extreme infectivity, acute onset, short duration, and sharp crisis. Recent investigations which have been carried out with a view to establish its bacteriological ætiology, and the results of which have been published in the *Lancet*, show that the prevailing illness is not true influenza due to Pfeiffer's bacillus, but is apparently a febrile condition resulting from the presence and activity of a diplococcus which has been isolated from the sputum, nose, and throat. Fortunately, while the outbreak has been widespread, the cases have been comparatively mild, although in London and elsewhere a number of deaths has been reported either from toxæmia or from pulmonary complications. The rapidity with which the disease has spread points to extreme susceptibility on the part of the population to attack by the specific organism, and to the presence of conditions such as dust, direct contact, etc., which favour the spread of infection. Prophylactic measures are uncertain, but efficient ventilation, quinine, and naso-pharyngeal antisepsis appear to give the best results.

### Sanatorium Administration.

An inquiry which has been held by the Local Government Board with reference to the administration of a sanatorium, in consequence of complaints made by a number of discharged soldiers, illustrates the difficulties which arise from time to time with regard to the control and administration of such institutions. Complaints made by sanatorium patients invariably relate to the question of food and to rules and restrictions. At the present time sanatorium and hospital authorities have a very difficult problem to face in providing a plentiful and varied diet, and they obviously cannot be blamed for the existence of circumstances over which they have no control. No doubt also the discharged soldier finds it difficult to accustom himself to those rules and restrictions which are considered necessary in every well-conducted sanatorium, or to appreciate their significance from his own individual standpoint. Any feeling of resentment in consequence of such restrictions will naturally magnify any cause for complaint which might exist. It is necessary, therefore, at the present time, with regard to sanatorium administration, that constant and careful attention should be given to the question of diet, and that all necessary rules and restrictions, while not relaxed, should be applied with knowledge, foresight, and tact.

### The Royal Sanitary Institute.

### RESULTS OF EXAMINATIONS.

The examinations of the Royal Sanitary Institute were held in London on July 26 and 27, when the following candidates were awarded certificates:—

Sanitary Science as Applied to Buildings.—Cross, Walter Edward (Dalston); Morris, Reginald James (Liverpool).

Maternity and Child Welfare.—Butcher, Mary (Drakefell Road, London); Ellis, Christine Bernard (Leicester); King, Constance (Gayton); Swiss, Emily Constance (Worksop); White, Anna Cornelia (Bristol).

Women Health Visitors and School Nurses.—Brown, Annie Gertrude (Wanstead); Dibdin, Marianne Stewart (Lingfield); Dyball, Florence Margaret Agnes (Norwich); Elliott, Ada Elizabeth (Lavender Hill); Gibbins, Rachael Jane (Edgbaston); Hill, Alice Emily Ellen (Portslade-by-Sea); Miller, Florence Maud (Battersea); Oakley, Annie (North Weald); Orpen, Angela Mary Kathleen (Bristol); Peacock, Florence Thornton (Islington); Penn, Florence A. (Leytonstone); Price, Mary Catherine (Bristol); Reed, Ethel Marjorie (Battersea Park); Robinson, Elizabeth Augusta (Dartmouth Park Hill); Stoddart, Alice Mary (Hackney); Thompson, Ina Margaret Isabel (Clapham Common); Todd, Edith Marjorie (New Romney); Ward, Agnes (Hillingdon Heath); Wells, Winifred Mary (Hammersmith); Wheeler, Alice Maud (Oxford).

School Hygiene.—Bambridge, Evelyn Maud Stacpoole (Canterbury); Banford, Sabina Mary (Wembley); Chown, Marianne Collingwood (St. Mark's Road, London); Collman, Marian Katharine (New Cross); Dryden, Alice Jean (Chelsea); Goulston, Hazel Mary (Denbigh Place, London); Low, Kate Marjorie (Brook Green); Penwill, Evelyn Victoria (Sutton); Pummell, Hilda Mary (Wimbledon); Robinson, Marjorie (Leeds); Walsh, Emmeline Nelson (Hammersmith).

Passed Part I. only:—

George, Gladys Dora (Weymouth); Perrott, Norah (Bromley); Wills, Edith Mellen (Denmark Hill).

### Matrons' and Sisters' Appointments.

Bermondsey Military Hospital.—Miss Margaret Watson has been appointed sister. She was trained at Croydon General Hospital, and has since worked as a sister under the Belgian and British Red Cross Societies.

Dudley, Guest Hospital.—Miss Evelyn M. Abbott has been appointed matron. She was trained at Plaistow Fever Hospital and the Royal Free Hospital, Gray's Inn Road, and was later pupil-housekeeper and temporary home sister at the Children's Hospital, Great Ormond Street, and assistant matron of the Royal Victoria and West Hants Hospital, Boscombe. Miss Abbott has also done military nursing both in England and France.

Ewell (County of London) War Hospital, Epsom.—Miss Douglas, Miss Colbourne, Miss Franklin, and Miss Bayliss have been appointed sisters. Miss Douglas was trained at Edinburgh Royal Infirmary, and has since been sister at Morningfield Hospital, Aberdeen; Miss Colbourne was trained at Ipswich, and has since been head sister at Wayland Military Hospital, Attleborough; Miss Franklin was trained at Bromley-le-Bow, and has since been a staff nurse of Queen Alexandra's Imperial Military Nursing Service Reserve; Miss Bayliss was trained at Croydon, and has since been sister at Tredegar Infirmary, Monmouthshire.

Haydock Cottage Hospital, St. Helens.—Miss Agnes Bone has been appointed matron. She was trained at St. Helens Borough Hospital and at the Royal Albert Edward Infirmary, Wigan, where she was later staff nurse, sister of the women's ward, the theatre, and the men's ward. Miss Bone has also done private nursing.

Home for Discharged Neurasthenic Sailors and Soldiers, Bray Court, Maidenhead (under the National Hospital, Queen Square).—Mrs. Emilie Drowley has been appointed matron-in-charge. She was trained at the East Sussex Hospital, Hastings, and at St. Leonards, and has since been sister-in-charge at the Nurses' Home, St. George's Hospital, and night sister at the Royal Sea Bathing Hospital, Margate.

Leicestershire County Council, Mowsley Sanatorium.—Miss Jean Cardwell Alcock has been appointed matron. She was trained at Crumpsall Infirmary, Manchester, and has since been matron of Hinckley Tuberculosis Residential Dispensary and sister-in-charge of Kimberworth Hospital for Consumption.

Nottingham General Hospital.—Miss E. Wheeldon has been appointed sister of the isolation block; Miss F. M. Rogers of the women's surgical ward; Miss Winifred Tooley, night sister; and Miss C. R. Bosankoe, sister. Miss Wheeldon was trained at Erdington Infirmary, Birmingham, and holds the certificate of the Central Midwives Board. She has since been holiday sister at Erdington Infirmary and out-patient theatre and night sister at Cheltenham General Hospital. Miss Rogers was trained at the London Hospital, Whitechapel, and has since been sister of the women's medical and surgical wards at Ancoats Hospital, Manchester, night sister at the Princess Alice Hospital, Eastbourne, and sister of the men's medical wards at the Bath United Hospital. Miss Tooley was trained at Derby Royal Infirmary, where she was later staff nurse, and has since been staff nurse in the military wards at Nottingham General Hospital and sister of the medical and surgical wards at the Royal West Sussex Hospital, Chichester. Miss Bosankoe was trained at the South Devon and East Cornwall Hospital, Plymouth, where she was later sister, and has since been sister of the women's surgical ward at Blackburn Royal Infirmary and of the women's and children's wards at Gravesend Hospital.

St. James' Infirmary, Balham.—Miss Charlotte Walker has been appointed ward sister. She was trained at Booth Hall Infirmary, Manchester, where she has since been staff nurse. She has also been staff nurse at Bradford War Hospital.

**NOTE.—Particulars of Appointments for insertion in this column will be welcomed.**

### Gifts and Bequests.

Mr. George Frederick Kendall, of Loxley Hall, Loxley, Stratford-on-Avon, left £250 to the Stratford-on-Avon General Hospital.

Mr. Henry Smith, of Brantingham, Yorks, left £200 each to Hull Royal Infirmary and the Beckett Barnsley Hospital and Dispensary.

Mr. Alfred Marsh, of Brierley Hill, left £300 each to the Corbett Hospital, Amblecote, and the Royal Orphanage, Wolverhampton.

The Royal Dental Hospital, Leicester Square, has received a donation of £500 from the Trustees of Smith's (Kensington Estate) Charity.

The Great Northern Central Hospital has received £500 from the trustees of Smith's (Kensington) Charity, and £100 from the National Sunday League.

The ultimate residue of the estate of Mrs. E. Dudley, of Edgbaston, will be distributed between a number of charities, including the General Institution for the Blind, Edgbaston, and the Queen's Hospital and the Hospital for Women, Birmingham.

After the death of Lady Alexander the residue of the estate of Sir George Alexander will be divided amongst the Actors' Benevolent Fund, the Actors' Orphanage, King Edward's Hospital Fund, and the Vicar of Chorley Wood for the poor of his parish.

Mrs. Gertrude Lorrilard, widow of Mr. J. Lorrilard, of New York, left estate to the gross value of £34,681; the residue is left in trust for her mother for life, and then £1,000 to the Cancer Hospital, and one-third of the balance, less some small legacies, for British charitable purposes.

To complete the allocation of £120,000 left for distribution by the late Mr. Leopold Salamons, the committee nominated have announced a list of donations, which include the following: £10,000 to the Infant Welfare Centre, £10,000 to Epsom College, £250 each to St. Peter's Hospital, Mother's Hostel, Epsom, the Royal Surgical Aid Society, and the Surgical Home for Boys, Banstead.

Mr. Henry Patteson Strong, of Purley, a gunner in the Royal Garrison Artillery, who was killed in action in France on October 29, 1917, left £1,000 to endow a bed in Guy's Hospital and £300 to Dunster Cottage Hospital.

Miss Emma Levy, of Gloucester Terrace, W., left £1,500 to the London Hospital for the endowment of a bed in the Goldsmid Female Ward in memory of her mother, Alicia Levy, and £50 each to the Surgical Aid Society, St. Mary's Hospital, the Hospital for Sick Children, and the Royal Hospital for Incurables.

Settles Street School, Commercial Road, has lately given nearly £100 to the London Hospital. Lord Knutsford attended to receive the cheque, which is the latest fruit of an annual collection. The total sum received from the school is now nearly £1,000, and its gifts in kind include toys, vegetables, and groceries.

Miss Louisa Sophia Scott, of Artillery Mansions, Victoria Street, S.W., and Bexhill-on-Sea, Sussex, bequeathed the residue of her estate, the gross value of which was £33,664, after payment of legacies of £2,000, in equal shares to the Hospital for Sick Children, Great Ormond Street, and the Royal Hospital for Incurables, at Putney.

